# Establishing reference intervals for electrolytes in newborns and infants using direct ISE analyzer

**DOI:** 10.1186/1756-0500-6-199

**Published:** 2013-05-20

**Authors:** Mulugeta Melkie, Mahilet Yigeremu, Paulos Nigussie, Tilahun Teka, Samuel Kinde

**Affiliations:** 1 Department of Medical Laboratory Science, Arbaminch University, Arbaminch, Ethiopia; 2 Faculty of Medicine, Addis Ababa University, Addis Ababa, Ethiopia; 3 Ethiopian Health and Nutrition Research Institute, Addis Ababa, Ethiopia

**Keywords:** Reference intervals, Electrolytes, Robust method, Newborns, Infants, Cord blood, Direct ISE

## Abstract

**Background:**

To generate clinically applicable reference intervals (RIs) for commonly requested electrolytes in Ethiopian newborns and infants that can help in early detection, close monitoring and correction of electrolyte abnormalities. Cord blood (from newborns, n = 60) and venous blood samples (from infants, n = 57) were collected and analyzed using direct ISE analyzer, AVL (9181). MedCalc® software was applied to determine the robust upper and lower end points covering 95% of the reference values of each electrolyte with respective 90% CIs.

**Findings:**

This is an extension report of our recent study; and hence is resulted from the same data source. The level of Na^+^ and K^+^ showed difference in newborns and infants even though combined RIs were suggested by the Haris and Boyd rule as 126–143 mmol/l and 4.0-7.9 mmol/l respectively. However, Cl^-^ values failed to show such a difference and thus a combined RI was determined to be 100–111 mmol/l. Almost all maternal, neonatal and infantile factors were not able to affect the values of the electrolytes.

**Conclusion:**

Combined RIs are suggested for the interpretation of electrolyte values in newborns and infants without taking the effect of maternal, neonatal and infantile factors into account. Since the RIs were different from previously reported values, it will be appropriate to apply such RIs for the interpretation of electrolyte values in Ethiopian pediatric population.

## Background

This work is an extension of the study that we have recently published [1]. A test for electrolytes mostly includes the measurement of sodium (Na^+^), potassium (K^+^) and chloride (Cl^-^) ions. Disturbance on the level of these electrolytes like hyponatremia, hypokalemia and metabolic acidosis are common in children with or without diarrhea and dehydration, especially in those with diarrhea superimposed up on malnutrition [2-4].

Hyponatremia is associated with an increased risk of mortality and prolonged hospitalization in sick children [5]. It is also the common cause of seizures in infants and is the leading cause of afebrile seizures in this population. In the treatment of hyponatremic seizures, correction of the electrolyte disturbance is more effective than using anticonvulsants [6]. Hypokalemia also occur frequently among pediatric patients in ICUs, in sick children especially in those with diarrhea and pneumonia [7,8].

Mostly, electrolyte abnormalities remain unrecognized resulting in mortality and morbidity in pediatric population [3,4]. Since the specific symptoms of electrolyte abnormality often merged with the underlying disease, close monitoring and correction of electrolyte abnormalities is important to reduce morbidity and mortality [9]. Hence, the aim of this study was to generate clinically applicable reference intervals (RIs) for commonly requested electrolytes (Na^+^, K^+^ and Cl^-^) in Ethiopian newborns and infants (≤ 1 year) so that timely recognition of disturbance in these analytes would be possible.

## Method and materials

A total of 117 newborns and infants in Tikur Anbessa Specialized Hospital (TASH) and Teklehaymanot Health Center (THC) respectively were included for this cross sectional study from November 2010 to April 2011. The inclusion and exclusion criteria and data collection and analysis were described in our recent publication [1]. Cord blood (from newborns) and venous blood samples (from infants) were collected and analyzed using direct ISE analyzer (AVL 9181, Roche diagnostics GmbH, Germany). Standard operating procedure was prepared and strictly adhered to during sample collection to avoid preanalytical errors (e.g. hemolysis). Samples with gross hemolysis were not included in this study.

Ethical clearance was obtained from the Department of Medical Laboratory Sciences research review committee and from the Institutional Review Board (IRB) of Addis Ababa University, Faculty of Medicine. Informed (written) consent was also obtained from mothers before specimen and data collection.

## Findings

### Analytical performance of the methods

Intra-assay coefficients of variations (CVs) for the three electrolytes were ≤ 2.30% in the three levels of the control sera (ISETROL Level 1, 2 and 3). Similarly, the inter-assay CVs were ≤ 2.24% for the three levels of the control sera (Table 1). Commercial quality control sera (ISETROL Level 1, 2 and 3) were included in every session of analyses. LJ charts were then plotted and all the quality control results were in the acceptable limits.

**Table 1:** **Intra- and inter assay CVs determined from duplicate analysis of QC materials ISETROL (LOT 9168**^†^, 9279^* ^**and 9391) for Na**^**+**^**, K**^**+ **^**and Cl**^**-**^

**Analytes**	**Intra assay CVs**			**Inter assay CVs**		
	**Level 1**^**†**^	Level 2^*^	**Level 3**^**§**^	**Level 1**^**†**^	Level 2^*^	**Level 3**^**§**^
Sodium	1.22%	1.16%	1.09%	2.11%	1.66%	1.54%
Potassium	1.04%	1.03%	2.06%	**2.24%**	2.01%	2.12%
Chloride	**2.30%**	1.36%	1.35%	1.18%	1.48%	2.00%

### Effect of neonatal, infantile and maternal factors on electrolytes

**Figure 1:**
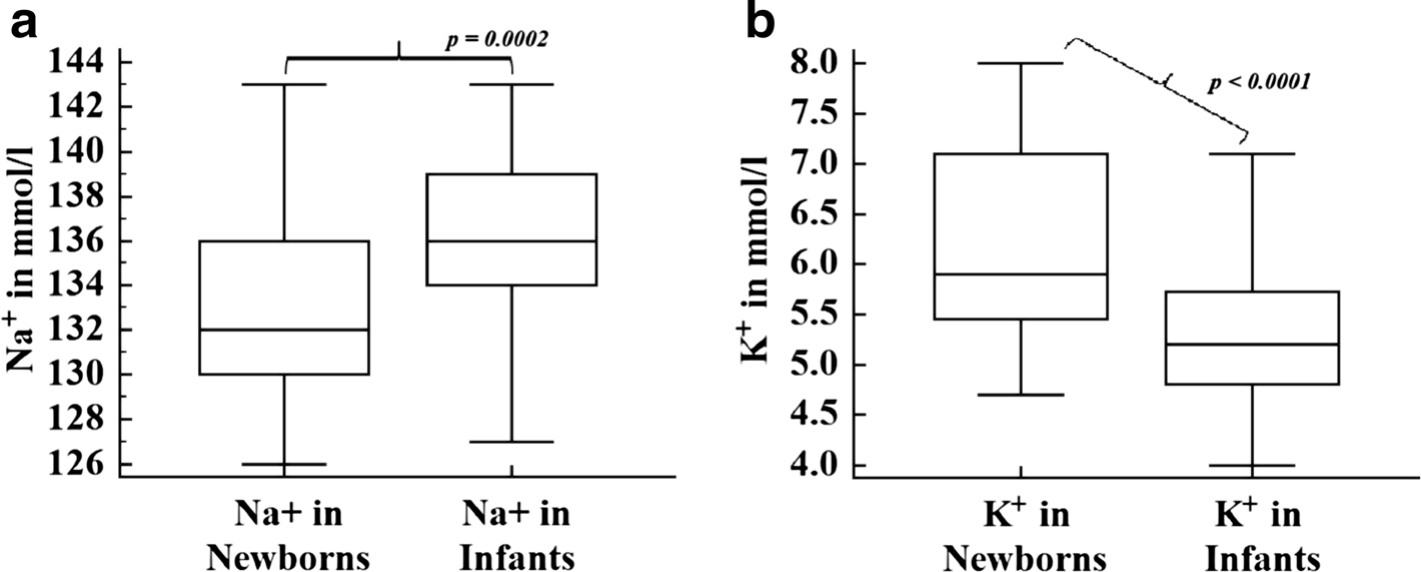
**Box and Whisker plots indicating the difference among newborns and infants in values of sodium (a) and potassium (b); [The box is 1**^**st **^**and 3**^**rd **^**quartiles while whiskers are lowest value, median and highest value].**

The demographic data about the mothers, infants and newborns has been stated in our recent report [1]. Except for Cl^-^ (*p = 0.052*), Na^+^ and K^+^ concentrations showed statistically significant difference in newborns and infants (Figure 1a and 1b). The effect of maternal and neonatal factors on the values of electrolytes is presented in Table 2. Similarly, the effect of infantile and maternal factors on the values of electrolytes is presented in Table 3.

**Table 2:** Effect of maternal and neonatal factors on the values of electrolytes in newborns

	**Variables**					
	**Sex**	**Mode of delivery**	**Maternal parity**	**Maternal education**	**Maternal occupation**	**Maternal alcohol consumption**
**Na**^**+**^	0.857*	0.633*	0.362*	0.836^†^	0.450^†^	0.687*
**K**^**+**^	0.412*	0.282*	0.909*	0.490^†^	0.442^†^	0.283*
**Cl**^**-**^	0.662*	0.490*	0.0006*	0.330^†^	0.148^†^	0.09*

* Independent sample *t*-test.

† ANOVA test.

**Table 3:** Effect of maternal and neonatal factors on the values of electrolytes in infants

	**Variables**					
	**Age group**	**Sex**	**Feeding practice**	**Maternal occupation**	**Maternal education**	**Maternal parity**
**Na**^**+**^	0.06*	0.73*	0.664^†^	0.935^†^	0.968^†^	0.674*
**K**^**+**^	0.27*	0.78*	0.709^†^	0.546^†^	0.482^†^	0.706*
**Cl**^**-**^	0.28*	0.51*	0.494^†^	0.380^†^	0.320^†^	0.445*

* Independent sample *t*-test.

† ANOVA test.

### RI Calculations for the electrolytes

As clearly depicted in Table 4, there were no outlier values detected in the three electrolytes. Similarly, the distributions were all Gaussian/normal. The RIs were then determined in newborns and infants. Harris and Boyd analysis suggested that partitioning should not be done and combined RIs should be used as the two RIs are in close proximity to each other.

**Table 4:** Summary of RI determinations of electrolytes (mmol/l) in newborns and infants

		**N**	**Outlier (Tukey)**	**Min. value**	**Max. value**	**Mean (95% CI)**	**DAP test**	**Lower limit (90% CI)**	**Upper limit (90% CI)**	**RI**	**Harris and Boyd**
**Na**^**+**^	**Newborns**	60	No	126	143	133.2	*P = 0.157*	124.3	141.4	**124-141**	***Combined RIs are suggested***
						(132.1-134.2)		(123.1-125.9)	(139.4-142.8)		
	**Infants**	57	No	127	143	136	*P = 0.129*	129	144.2	**129-144**	
						(135–137)		(127.1-130.5)	(142.6-145.5)		
	**Combined**	117	No	126	143	134.5	*P = 0.327*	126	143	**126-143**	
						(134–135)		(126–127)	(142–143)		
**K**^**+**^	**Newborns**	60	No	4.7	8.0	6.20	*P = 0.108*	3.99	8.26	**4.0-8.3**	***Combined RIs are suggested***
						(5.94-6.46)		(3.63-4.33)	(7.72-8.63)		
	**Infants**	57	No	4.0	7.0	5.32	*P = 0.090*	3.66	6.72	**3.7-6.7**	
						(5.12-5.52)		(3.37-3.98)	(6.39-7.07)		
	**Combined**	117	No	4.0	8.0	5.69	*P = 0.118*	4.01	7.9	**4.0-7.9**	
						(5.52-5.87)		(3.95-4.1)	(7.52-8.04)		
**Cl**^**-**^	**Newborns**	60	No	100	115	105.9	*P = 0.748*	99.6	112.2	**100-112**	***Combined RIs are suggested***
						(105.1-106.7)		(98.4-100.8)	(111–113.3)		
	**Infants**	57	No	100	110	104.9	*P = 0.536*	99.4	110.2	**99-110**	
						(104.2-105.6)		(98.5-100.3)	(109.2-111.2)		
	**Combined**	117	No	100	111	105.3	*P = 0.39*	99.7	111	**100-111**	
						(104.8-105.8)		(99.4-100)	(110.2-111.3)		

## Discussion

According to this study, Na^+^ showed statistically significant increment in infants than in newborns. However, according to the Harris and Boyd rule, the RIs in newborns and infants were not far apart enough to be used independently. So that, combined RI are suggested for interpretation of Na^+^ levels in both groups (RI = 126-143 mmol/l). The decreased level of Na^+^ in newborns can be explained by continuous Na^+^ excretion as a result of decrement in the amount of total body water in the fetus with advancing gestational age [10]. The excretion of Na^+^ also continues immediately after birth through urine, sweat and feces as a result of the mandatory water loss during transition from in-utero to ex-utero environment. But, several days after birth, fluid and electrolyte requirements increase as the infant starts to grow [11].

On the contrary, K^+^ level was higher in newborns than in infants without requiring for a separated RI (RI = 4.0-7.9 mmol/l). Our finding is supported by a previous study which indicated that K^+^ has age dependent variability showing high results in the newborns and then declining to reach adult level by the age of 3 [12]. But, Cl^-^ values failed to show such a difference and thus a combined RI was determined to be 100–111 mmol/l.

Generally, both the upper and lower limits of the RIs of electrolytes determined in this study were different from previously published values, text book values and kit insert values for other target populations of similar age group [13-16] and from adult values [17]. Especially, the upper limit of K^+^ was higher and the lower limit of Na^+^ was lower (Table 5). These findings strengthened our recent report on the deviation of previously established RIs for Liver Function Test (LFT) analytes from the ones established for Ethiopian pediatric population [1].

**Table 5:** Comparing the RIs of electrolytes in newborns and infants with values for the same age group and for adults in previous studies, text books and kit inserts

	**Na**^**+ **^**(mmol/l)**	**K**^**+ **^**(mmol/l)**	**Cl**^**- **^**(mmol/l)**
**Current study**	Combined = 126-143	Combined = 4.0-7.9	Combined = 100-111
**Perkins et al.**[13]	venous cord blood = 135-143	venous cord blood = 3.8-6.8	venous cord blood = 102-112
**Text books**	Newborns = 133–146 [14]	Newborns = 4.5-7.2 [15]	<1 year = 96–111 [15]
	Children	Children = 3.5-5.8 [15]	1-17 years = 102–112 [14]
	and adults = 135–148 [14]	Adults = 3.5-5.5 [15]	Adults = 100–108 [14]
**Kit inserts**	136-145 [16]	3.5-5.1 [16]	97-111 [16]
**Adult values**	141.4-152.5 [17]	3.9-5.8 [17]	100.5-111.7 [17]

This study revealed that except maternal parity, the remaining neonatal and maternal factors failed to affect the values of electrolytes in newborns. The chloride level in newborns from primiparous mothers was significantly higher than newborns from multiparous mothers (*p = 0.0006*). But, the increment was not enough to dictate establishment of separated RIs. Our finding is in contradiction with a previous study that reported no association in between maternal parity and maternal electrolyte levels [18]. Thus, we recommend further investigation concerning the impact of parity on fetal electrolytes levels particularly of Cl^-^ levels.

In our study, we did not found any effect imposed by maternal and infantile factors on the three electrolyte levels in infants.

## Conclusion

From this study, we can conclude that combined RIs are suggested for the interpretation of electrolyte values in newborns and infants without taking the effect of maternal, neonatal and infantile factors into account. Moreover, the RIs were different from previously reported values for other target population of similar age group, kit insert values and adult values. Hence, it will be better to apply such RIs for the interpretation of electrolyte values in Ethiopian pediatric population.

## Competing interests

The authors declared no competing interests in this research. In fact, the research was financially supported by Addis Ababa University; and other non-financial supports were also obtained from Mesroy international plc (Reagents) and Medcalc Software Company (statistical software).

## Authors’ contributions

MM, TT and SK have participated in the conception and design of the study. MY and TT have participated in the selection of study participants. MM and PN have participated in the laboratory analysis and acquisition of data. MM, TT, MY and SK have participated in preparing and critically reviewing the draft manuscript. All authors have read and approved the final manuscript.
